# DHA Supplementation during Pregnancy in Women with Obesity Normalizes IGF2R Levels in the Placenta of Male Newborns

**DOI:** 10.1155/2023/1515033

**Published:** 2023-06-27

**Authors:** Juan José Castro, Adriana Umana-Perez, Erika Castaño-Moreno, Paola Casanello, Ana María Ronco

**Affiliations:** ^1^Departamento de Química, Facultad de Ciencias, Grupo de Investigación en Hormonas, Universidad Nacional de Colombia, Código Postal: 111321, Bogotá, Colombia; ^2^Laboratory of Nutrition and Metabolic Regulation, Human Nutrition Unit, Institute of Nutrition and Food Technology, Doctor Fernando Monckeberg Barros (INTA), University of Chile, Post Code 7830490, Santiago, Chile; ^3^Institute for Obesity Research, Tecnologico de Monterrey, Ave. Eugenio Garza Sada 2501, Monterrey 64849, NL, Mexico; ^4^Department of Neonatology and Department of Obstetrics, School of Medicine, Pontificia Universidad Católica de Chile, Post Code: 8330024, Santiago, Chile

## Abstract

**Introduction:**

Insulin-like growth factor receptor 2 (IGF2R) regulates placental nutrient transport, and its soluble form is related to obesity in adults. If the placental expression of IGF2R is altered in women with obesity is unknown. Whether maternal supplementation with docosahexaenoic acid (DHA), a polyunsaturated fatty acid with anti-inflammatory properties, has a modulatory role in IGF2R's function has not been elucidated. We hypothesized that maternal obesity (Ob) would be associated with alterations in placental IGF2R expression, which may be prevented with DHA supplementation during pregnancy.

**Methods:**

At delivery, we obtained placentas from women with Ob (BMI ≥ 30 kg/m^2^, *n* = 17), Ob supplemented with 800 mg/day of DHA during pregnancy (Ob + DHA, *n* = 13), and normal-weight women (Nw, BMI ≥ 18.5 ≤ 24.9 kg/m^2^, *n* = 14). The IGF2R mRNA and protein were determined by RT-PCR and western blotting, respectively. Moreover, we quantified the gene expression of molecules that modulate the IGF2R function in the extracellular domain, such as TACE/ADAM17, PLAU, and IGF2. Mann–Whitney and Kruskal–Wallis nonparametric tests were used to compare results between two or three groups accordingly.

**Results:**

The IGF2R levels in the Ob placentas of the male offspring were higher than in the Nw group. The DHA supplementation prevented this effect, suggesting an unknown relationship between IGF2R-Ob-DHA in placental tissues.

**Conclusion:**

We report, for the first time, that DHA supplementation during pregnancy in women with obesity normalizes the increased IGF2R levels in male placentas, reducing the risk of adverse outcomes related to the IGF2/IGF2R system in male newborns.

## 1. Introduction

Insulin-like growth factors (IGFs) have a potent mitogenic activity and appear to be major determinants of fetal growth [1, 2]. These factors are expressed in the fetus and placenta [1, 3], IGF2 being the most abundant and active participant in early embryogenesis and during fetal development [1, 4]. This ligand binds to the cell surface tyrosine kinase receptors [4, 5], leading to the endogenous autophosphorylation of tyrosine residues followed by the phosphorylation of downstream signaling pathways: the mitogen-activated protein kinase (MAPK) and the phosphoinositide-3 kinase (PI3K)/AKT pathway [6, 7]. Upon activation, these downstream molecules mediate a myriad of intracellular signals, including the regulation of glucose transport, protein synthesis, cell proliferation, migration, and survival [4, 7, 8].

**Figure 1:**
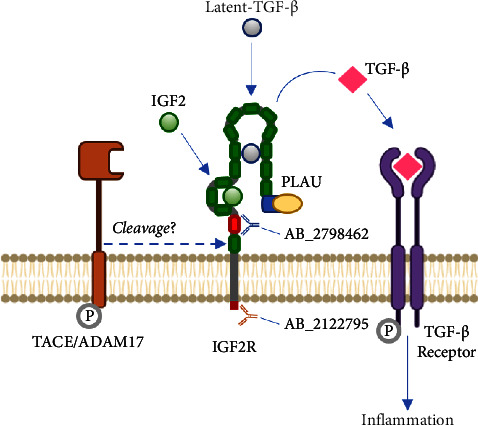
IGF2R activation and signaling. IGF2R interacts with PLAU in its domain 1 and with the latent (inactive) form of TGF-*β*, which would facilitate its activation. Once TGF-*β* is activated and interacting with its receptor, it will allow the phosphorylation of TACE/ADAM17 through the MAPK pathway, translocating it to the cell membrane to allow the cleavage of the IGF2R receptor. Specific sites of interaction of both antibodies used in this study are shown. AB_2798462 is addressed against the extracellular domain, and AB_2122795 is addressed against the cytoplasmic domain of IGF2R.

The insulin-like growth factor type 2 receptor (IGF2R) is a glycoprotein of nearly 300 kDa expressed in most tissues, including the placenta. This receptor has a great affinity for IGF2 as well as for molecules with a mannose 6-phosphate (M6P) structure, e.g., prorenin, latent-transforming growth factor-beta (TGF-*β*) [9, 10]. IGF2R interacts with the plasminogen-activated urokinase receptor (PLAUR), which, together with the urokinase-type plasminogen activator (PLAU), promotes plasminogen activation to plasmin. This allows the activation of TGF-*β* [11], a proinflammatory cytokine which, along with other cytokines, is increased in placentas with a high accumulation of lipids, inflammation, and oxidative stress [12, 13], such as those from women with obesity [14, 15]. TGF-*β* regulates its activity by the negative feedback of its signaling mechanisms through a MAPK-mediated activation of a TACE/ADAM17 metalloprotease. Finally, TACE/ADAM17 is recruited to the cell membrane leading to the cleavage of the IGF2R receptor [16, 17] (Figure 1).

IGF2 and IGF2R contribute to placental development and nutrient transport, indirectly impacting fetal size [18, 19]. The molar ratio of IGF2/sIGF2R (soluble form of the receptor ∼220 kDa) is directly related to placental and newborn weights [20], and there is a direct relationship between sIGF2R levels and gestational age in normal pregnancies and preeclampsia [21]. It has been recently shown that the mice imprinted IGF2/IGF2R axis is critical for matching placental microvascular expansion to fetal growth [22]. Consequently, an altered function of this hormone signal may affect trophoblast morphogenesis and placental function, increasing the risk of developing pregnancy-associated diseases.

IGF2 levels, on the other hand, increase dramatically during fetal adipogenesis, so the autocrine and paracrine effects of this ligand are critical mechanisms controlling the accumulation and metabolism of lipids, adipose tissue growth, and differentiation [23, 24]. Remarkably, the plasma content of sIGF2R is elevated in adult patients with morbid obesity and type 2 diabetes. However, if these patients undergo gastric banding surgery, the levels of this receptor decrease, suggesting a direct, although unclear, association between this form of the receptor, the ligand, and this clinical condition [25]. The plasma levels of sIGF2R and body mass index (BMI) have been positively correlated with a regulatory mechanism at the nutritional level [21, 25, 26].

Considering this, it is relevant to understand the biological mechanisms involved in the human placental IGF2- IGF2R system and its role in fetal development, especially in maternal obesity. This primary global health concern has rapidly increased, reaching pandemic proportions [27]. In particular, the prevalence of maternal obesity in Chile has grown dramatically, with more than 70% of pregnant women showing some degree of overweight or obesity (BMI ≥ 25 kg/m^2^ and BMI ≥ 30 kg/m^2^, respectively) [28, 29]. This condition has been associated with an increased risk of high neonatal birth weight, increased adipose tissue during fetal life, insulin resistance, and an increased risk of developing degenerative diseases in adulthood due to epigenetic programming during development [30, 31]. Likewise, preeclampsia, hypertension, and maternal obesity can cause the dysregulation of placental function, triggering elevated expression of inflammatory markers, hypoxia, chronic villitis, fetal thrombosis, and villous edema, with marked sexual dimorphism [32–34].

In the search for new treatments that control or diminish the effects of chronic inflammation in obesity, the use of polyunsaturated fatty acids such as docosahexaenoic acid (DHA) has been proposed. In a recent study, DHA supplementation in overweight/obese pregnant women reduced inflammation markers in the placenta and the adipose tissue of pregnant women [35].

Therefore, this study aimed to evaluate if maternal obesity is associated with changes in the placental expression of IGF2R and its associated molecular components at the extracellular domain, such as PLAU and TACE/ADAM17, and whether DHA supplementation during pregnancy modifies this expression and its downstream signaling. The need to investigate and understand the effects of this maternal pathology is of great importance for public health due to the increased obstetric complications of this condition.

## 2. Methods

### 2.1. Sample Collection

Term placentas from healthy women with single-term pregnancies (37 to 41 weeks of gestation) were collected immediately after delivery. Placentas were separated into three groups, those extracted from Ob women (Ob) (BMI ≥ 30 kg/m^2^, Ob: *n* = 17, males = 10 and females = 7), a group from Ob women supplemented with 800 mg/day of DHA throughout the pregnancy period (Ob + DHA) (BMI ≥ 30 kg/m^2^, Ob: *n* = 13, males = 6 and females = 7), and those from normal-weight women (Nw) (BMI ≥ 18.5 ≤ 24.9 kg/m^2^, Nw: *n* = 14, males = 7 and females = 7). Ob + DHA group received oral supplementation as capsular preparations (4 capsules of 200 mg/day of DHA) based on *Schizochytrium* oil (S-oil, provided by DSM Nutritional Products, Kaiseraugst, Switzerland) during pregnancy (<15 weeks of gestation until delivery). The adherence to DHA supplementation was 50–55%, as described by Haghiac et al. [35]. Patients who consumed 75% of the capsules were the adherent population to the DHA supplementation. Exclusion criteria included maternal hypertension, diabetes, preeclampsia, smoking (>5 cigarettes/day), alcohol or drug consumption, multiple pregnancies, and genetic disorders in the newborn. The Ethics Committees from the South-East Metropolitan Health Service, the Institute of Nutrition and Food Technology (INTA) of the University of Chile, and the Pontificia Universidad Católica de Chile approved this protocol. The samples of this study were from patients participating in the Maternal obesIty/overweight control throuGh Healthy nuTrition (MIGHT) study [35]. All participants in this study signed informed consent.

Placentas were weighed after the cord and membranes were trimmed, immediately placed on ice, and dissected. The chorionic plate, chorioamniotic membranes, and decidua were removed [36]. The central trophoblast layer from at least five evenly distributed areas of the trophoblast plate was cut into small pieces of 3 × 3 mm^2^ (approximately 200 mg of tissue) and washed 3 three times with cold phosphate-buffered saline (PBS) [36]. The tissue for mRNA extraction was included in RNAlater™ (Invitrogen, USA) overnight and then stored at −80°C until analysis.

### 2.2. RNA Extraction, cDNA Synthesis, and Real-Time PCR

The total RNA of placental tissues was extracted with Trizol™ Reagent (Invitrogen, Carlsbad, CA), and RNA was kept in aliquots at −80°C. The RNA integrity of each sample was evaluated by a 260/280 ratio of 1.7–1.9 and by electrophoresis in agarose gels. Following the manufacturer's instructions, one microgram of mRNA was used to synthesize cDNA with the M-MLV kit reverse transcriptase (RT, Promega, Wisconsin, USA). ADAM17, IGF2, IGF2R, and PLAU mRNA levels were measured by the Eco qPCR System (Illumina San Diego, CA, U.S.A). Results were analyzed by Eco Real-time PCR System Software v4.1 (Illumina) and calculated by the Livak method [37] following the MIQE Guidelines [38]. The results were expressed as a reference to the geometric mean expression of two reference genes (GAPDH and YWHAZ) [39]. The results were referred to the Nw group as the control. The sequence of the gene primers is described in Supplementary Table 1.

### 2.3. Protein Extraction and Western Blot

Frozen placental explants (200 mg) of all groups were macerated in dry ice. Proteins were extracted with lysis buffer (Invitrogen, Carlsbad, CA) containing 0.5% Triton X-100 and protease inhibitor cocktail (Roche Diagnostic) and frozen at −80°C. Protein concentrations were determined by the Pierce® BCA Protein Kit Assay (Thermo Scientific, USA) using a standard curve of bovine serum albumin (BSA, 1.0 mg/mL). Total protein extracts were separated by electrophoresis in 6%-polyacrylamide gels and were electrotransferred to a PVDF membrane at 60 mA overnight. All membranes showed similar transfer after staining with Ponceau red. The membranes were blocked with 5% skimmed milk in Tween-TBS 0.05% (TTBS) for 1 h, washed with TTBS, and incubated with specific antibodies to detect IGF2R (300 kDa). Blots were incubated with anti-IGF2R (diluted 1 : 500, Cell Signaling #14364, RRID: AB_2798462, addressed to the extracellular domain, Figure 1) or anti-IGF2R (diluted 1 : 500, Santa Cruz Biotechnology sc-25462, RRID: AB_2122795, addressed to the cytoplasmatic domain, Figure 1) for 2 h at room temperature. Subsequently, the blots were incubated with an anti-rabbit antibody (diluted 1 : 5000, Rockland Antibodies and Assays–611–1302, RRID: AB_219720) for 1 h at room temperature. The anti-*β*-actin antibody (Sigma-Aldrich–A1978, diluted 1 : 5000, RRID: AB_476692) was used as a loading control and incubated for 2 h at room temperature under shaking. Blots were then incubated with a secondary anti-mouse antibody (Rockland Antibodies and assays 610–1319, diluted 1 : 5000, RRID: AB_219659) for 1h at room temperature under stirring. The blots were visualized and quantified in duplicate with Westar reagent chemiluminescent Supernova *η* Substrate using Western Blot Kit Cyanagen using a 510 BioSpectrum Multispectral Imaging System and quantified by UVP Framework in the WorksLS Vision software.

### 2.4. Statistical Analysis

**Figure 2:**
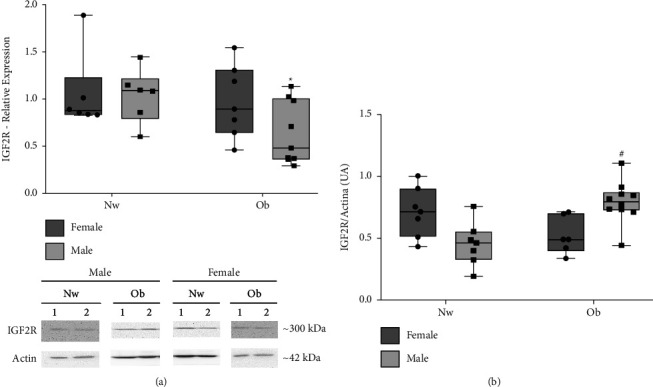
Expression of IGF2R in human placentas of Nw and Ob groups according to newborn sex. (a) IGF2R mRNA determined by RT-qPCR in relation to the geometric mean expression of 2 housekeeping genes (GAPDH and YWHAZ). (b) IGF2R protein content determined by WB using AB_2798462 and *β*-actin as calibrator. Western blot signals were determined by densitometry. Blots show representative samples of both groups. All results are expressed as median and 25^th^–75^th^ percentiles. In (a) Nw: normal-weight*n* = 12 (6 males and 6 females); Ob: obesity *n* = 16 (9 males and 7 females), ^*∗*^*p* < 0.05 Ob males vs. Nw males. In (b) Nw *n* = 14 (7 males and 7 females); Ob *n* = 17 (10 males and 7 females), ^#^*p* < 0.05 Ob males vs. Nw males, Mann–Whitney test.

**Figure 3:**
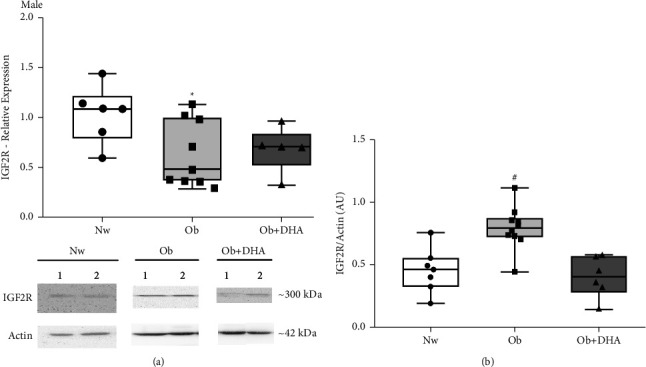
Expression of IGF2R in male placentas of Nw, Ob, and Ob + DHA groups. (a) Relative expression of IGF2R determined by RT-qPCR. (b) IGF2R protein content determined by WB using AB_2798462 and *β*-actin as calibrator. Blots show representative samples of the three groups. Results are expressed as median and 25^th–^75^th^ percentiles. In (a) Nw: normal-weight*n* = 6; Ob: obesity *n* = 9; Ob + DHA: obesity supplemented with DHA *n* = 5, ^*∗*^*p* < 0.05 Ob vs. Nw. In (b) Nw *n* = 7; Ob *n* = 10; Ob + DHA: *n* = 6, ^#^*p* < 0.01 Ob vs. Nw and Ob + DHA, Kruskal-Wallis test.

**Figure 4:**
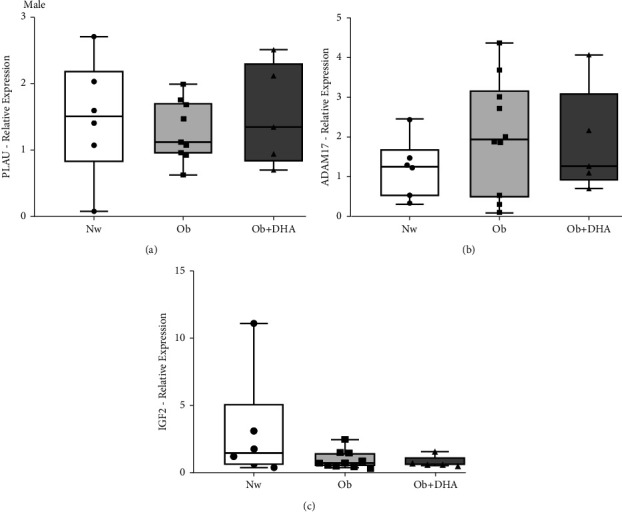
Relative gene expression of PLAU, ADAM17, and IGF2 in male placentas of Nw, Ob, and Ob + DHA groups. (a) PLAU mRNA, (b) TACE/ADAM17 mRNA, (c) IGF2 mRNA. Results were determined by RT-qPCR and expressed as median and 25^th^–75^th^ percentiles. For all the genes: Nw: normal-weight*n* = 6; Ob: obesity *n* = 10; Ob + DHA: obesity supplemented with DHA *n* = 5, Kruskal-Wallis test.

All results are expressed as median (25^th^–75^th^ percentiles).The nonparametric Mann–Whitney test was used to compare two groups (Nw and Ob, Figures 2(a) and 2(b)), and the Kruskal–Wallis test was used to compare three groups (Nw, Ob, and Ob + DHA, Figures 3(a) and 3(b), 4(a)–4(c)). Differences with *p* < 0.05 were considered significant. The statistical analysis was performed using GraphPad Software Inc. Version 7.

## 3. Results

The anthropometric characteristics of the newborns and their mothers, whose placenta samples were analyzed in this study, are presented in Table 1. As expected, pregestational weight and BMI in the Ob and Ob + DHA groups were higher compared to the Nw group. However, no differences were observed in the Ob vs Ob + DHA groups in all analyzed characteristics. There was also no difference in maternal weight gain during the pregnancy period between three groups. Concerning newborn characteristics, there were no differences in fetal weight and size or Ponderal Index at birth between groups.

### 3.1. IGF2R Expression (mRNA and Protein)

We first evaluated whether the obesity condition affects the placental IGF2R expression (mRNA and protein). Then, we determined the IGF2R mRNA in the placentas of the Ob and Nw women according to the newborn sex. As shown in Figure 2(a), the IGF2R mRNA of the Ob group was lower in the male compared to the Nw; however, no significant effect was demonstrated in the IGF2R mRNA in the female placentas of the two groups. Protein content was first analyzed using an antibody against the receptor's extracellular domain. Results in Figure 2(b) show that in the Ob group, IGF2R levels were higher than in the Nw group, although this effect was only observed in the male placentas. Since we found differences only in male placentas between the Ob and Nw groups, the results focus on this group.

When comparing the male placentas IGF2R mRNA between the three groups, we observed that the IGF2R mRNA in the Ob + DHA group did not decrease as the Ob group and tended to be similar to Nw, although results were not significant (Figure 3(a)). Regarding the protein levels, we observed that maternal DHA supplementation prevented the IGF2R increase observed in the Ob group (Figure 3(b)).

To explore whether the higher IGF2R content in male placentas of the Ob group was specifically related to the external molecular components, we determined the receptor protein content using the IGF2R antibody against the cytoplasmatic domain of the receptor. Results did not show any difference between Ob and Nw (Supplementary Figure 1), suggesting that the increased content of IGF2R observed in male placentas delivered by Ob women may be related to the external domain of the receptor.

### 3.2. IGF2, PLAU, and ADAM17 Expressions

The placental gene expression of PLAU, ADAM17, and IGF2 did not change between groups (Figures 4(a)–4(c), respectively).

## 4. Discussion

In this study, we found a lower placental expression of IGF2R in the Ob group compared to the Nw group, but this effect was only observed in male placentas. On the contrary, in the Ob group, IGF2R protein levels were higher than the Nw, and this effect was only observed in male placentas. This opposite effect between the gene expression and the protein levels may suggest the downregulation of the gene expression induced by the increased levels of IGF2R affected by obesity. In addition, we observed that the maternal DHA supplementation prevented the increase of the levels of IGF2R induced by maternal obesity in male placentas. To our knowledge, this is the first study reporting placental IGF2R expression in women with Ob and the effect of a DHA supplementation during pregnancy on the expression of this receptor.

The first approach of our study was to evaluate the anthropometric parameters of the subjects. The fetal anthropometrics, including weight, length, and Ponderal Index, did not show differences between groups. Maternal obesity mainly affects fetal adiposity and metabolic programming, which does not necessarily affect fetal weight. Maternal weight in women with Ob and excessive weight gain during pregnancy could increase fetal anthropometrics [40–42]. However, in this study, maternal weight gain during pregnancy was similar in both groups, as was gestational age at birth. Only the prepregnancy weight and BMI of the mothers (Ob vs Nw) were different. However, these differences did not affect the parameters of the newborns (weight, height), suggesting unaltered placental transfer of nutrients, evidenced by the similar placental weight between both groups [6, 43, 44] (Table 1). The range of gestational weight gain in the groups analyzed was higher than those recommended [44–46]. However, weight gain in pregnancy depends on multiple variables such as maternal lifestyle, ethnicity or socioeconomic factors, among others [40, 44–46]. These factors could lead to inconsistencies when associating them with clinical characteristics [47], as in this study. Likewise, other studies with maternal DHA supplementation in Ob women do not show changes in anthropometric data or weight gain compared to nonsupplemented women [47–49]. As previously shown, a decreased placental inflammation and differential modulation of placental nutrient transport capacity by DHA could underpin these effects [43], possibly mitigating the adverse effects of maternal obesity on the placental function. In addition, no changes were observed with maternal DHA supplementation on the newborn anthropometrics, as shown in previous studies, demonstrating that IGF2 and IGF2R expression in the placenta could not correlate with the neonatal size [22].

IGF2 is necessary for adequate human growth, and its overexpression is associated with fetal overgrowth and may play a role in the intrauterine programming of adipose tissue [50]. Therefore, the placental expression of its receptor, IGF2R, should be relevant to the biological function of IGF2 and its involvement in the risk of developing obesity in the offspring of women with obesity [19, 21].

The sexual dimorphism observed in this study has been previously evidenced under different conditions. Some studies suggest fetal sex-related differences in the expression profile of genes regulating inflammatory processes, immunotolerance, growth factors, nutrient transporters, or the maintenance of pregnancy [51, 52]. However, there are contradictory results [32, 51]. Although some models describe that female placental growth is slower than males [32], this is more demanding regarding metabolic and vascular adaptation, establishing an increased transport of nutrients to the fetus [52, 53].

Obesity is characterized by a chronic low-grade inflammatory state due to increased adipose tissue mass and enhanced production of proinflammatory mediators [54, 55]. This condition favors insulin resistance, a phenomenon described in other diseases, such as type 2 diabetes, systemic hypertension, dyslipidemia, and cardiovascular diseases [54, 55]. Similarly, gestational obesity leads to obstetrical complications such as gestational diabetes and hypertension, preeclampsia, fetal growth restriction, premature birth, macrosomia, and cesarean section, increasing the mortality and morbidity rate for both the newborn and the mother [56, 57].

In the presence of maternal complications, such as maternal obesity, the male placental strategy counteracts this event with few genetic, protein, and functional changes, thus ensuring continued growth but with suboptimal growth conditions [51, 52, 58]. Nevertheless, female placentas respond to multiple genetic and protein modifications, attenuating growth but ensuring survival [51, 52], which would partially explain the absence of changes in female placentas delivered by mothers of the Ob group.

Interestingly, we found that in male placentas delivered by Ob women supplemented with DHA during pregnancy, the IGF2R levels decreased to values comparable to those found in the placentas of normal-weight mothers. However, a DHA effect on the IGF2R mRNA of the Ob male placentas could not be proven, probably due to a low sample size. A recent report showed that in mice, a DHA enrichment diet reversed the sex-specific decrease in the male placenta and embryo weight following early prenatal stress [59].

Since the level of IGF2R receptors in the placentas was different in Ob women, as detected when using antibodies recognizing the amino acids surrounding Ala1675, localized in the extracellular region of the protein, and above the cleavage level, we suggest that the increased content in the placental IGF2R of Ob women may be due to a lower rate of cleavage, thus increasing the levels of active TGF-*β* and thus, favoring a chronic inflammation process characteristic of the obesity condition. This can be evidence because we did not find any difference in the content of IGF2R when we used an antibody that recognizes the cytoplasmic region (Figure Supplementary 1), suggesting that these changes occur at the extracellular binding site level.

It has been shown that about 90% of the IGF2R is found at the intracellular level, the Golgi apparatus or the endosomal lysosomal compartment, presumably participating in recognizing, classifying, and arranging the newly modified lysosomal enzymes in the trans-Golgi network [60]. Therefore, this difference would only occur at the cell membrane level.

The TACE/ADAM17 metallopeptidase can mediate the release of the extracellular region of IGF2R, playing a key role in modulating the growth effect. Furthermore, it could regulate the angiogenesis and the plasminogen-induced inflammatory process [16, 21]. We suggest that in response to this obesity condition, there would be a deregulation or absence of changes in its expression, activation, content, translocation, or perhaps an increased inhibition [61] in the trophoblast of Ob women. The lack of differences in the gene expression of placental ADAM17, PLAU, and IGF2 between the three groups suggests that these genes would not be affected by obesity.

Furthermore, IGF2R is involved in removing extracellular IGF2, internalizing it for degradation [62], and regulating its function. Remarkably, IGF2 and its signaling molecules promote structural changes in the placenta, increasing the volume and surface area of nutrient exchange in the hemochorial zone, particularly in term placentas. Therefore, they participate in a higher activation of the glucose transporters and in the A and L amino acid transport systems, allowing increased fetal growth and weight [63]. As mentioned earlier, there is a direct relation between the soluble form of the receptor and gestational age [21], indicating that the receptor must be cleaved by ADAM17/TACE, allowing fetal weight gain induced by IGF2. Our results suggest that placental IGF2R is less cleaved in women with Ob. This may explain why obstetric complications such as preeclampsia or fetal growth restriction are more frequent in male fetuses from women with obesity [33, 53]. However, these questions are open and need to be further addressed.

## 5. Conclusion

The male placentas of women with Ob present an increased protein content of IGF2R, and maternal supplementation with DHA throughout pregnancy prevented that increase. These results could explain the association of the expression of this receptor and its signaling with the deleterious effects of maternal obesity and the development of obstetrical complications in pregnancies with male fetuses.

## Figures and Tables

**Table 1:** Characteristics of mothers and newborns.

Characteristic	Normal weight (Nw)			Obesity (Ob)			Obesity + DHA (Ob + DHA)		
	Total *n* = 14	Males *n* = 7	Females *n* = 7	Total *n* = 17	Males *n* = 10	Females *n* = 7	Total *n* = 13	Males *n* = 6	Females *n* = 7
Pregestational weight (kg)	57.53 ± 2.48	57.88 ± 3.09	57.13 ± 1.70	84.86 ± 9.99^a^	86.87 ± 10.07^a^	82.29 ± 10.01^a^	88.48 ± 10.48^b^	86.36 ± 13.52^b^	90.60 ± 6.68^b^
Pregestational BMI (kg/m^2^)	22.62 ± 1.34	22.94 ± 1.05	22.26 ± 1.63	33.29 ± 3.44^a^	34.42 ± 3.84^a^	32.00 ± 2.60^a^	34.54 ± 2.82^b^	33.83 ± 3.19^b^	35.14 ± 2.54^b^
Weight at delivery (kg)	70.22 ± 3.65	70.41 ± 3.20	70.00 ± 4.37	91.76 ± 11.45^a^	91.93 ± 12.79^a^	91.57 ± 10.72^a^	96.04 ± 9.05^b^	97.29 ± 11.57^b^	94.79 ± 6.34^b^
BMI at delivery (kg/m^2^)	27.69 ± 2.75	27.99 ± 2.26	27.35 ± 3.37	36.42 ± 3.72^a^	37.24 ± 4.76^a^	35.60 ± 2.40^a^	37.29 ± 2.21^b^	37.94 ± 2.85^b^	36.72 ± 1.49^b^
Weight gain (kg)	12.21 ± 3.94	11.63 ± 3.29	12.87 ± 4.75	9.47 ± 4.00	9.64 ± 5.18	9.29 ± 2.43	8.87 ± 4.32	10.93 ± 3.97	6.00 ± 3.16
Gestational age (weeks)	39.11 ± 0.86	38.95 ± 0.88	39.30 ± 0.87	39.27 ± 1.14	39.03 ± 1.31	39.62 ± 0.82	39.00 ± 1.11	39.00 ± 1.41	39.00 ± 0.82
Newborn's weight (g)	3403.33 ± 331.63	3407.50 ± 293.34	3398.57 ± 395.20	3485.81 ± 553.46	3585.56 ± 700.48	3357.57 ± 278.33	3487.86 ± 400.29	3589.29 ± 404.84	3386.43 ± 399.09
Newborn's height (cm)	50.33 ± 1.72	50.50 ± 1.69	50.14 ± 1.86	50.16 ± 1.95	50.72 ± 2.20	49.43 ± 1.40	50.54 ± 2.03	50.36 ± 1.97	50.73 ± 2.23
Ponderal Index	2.67 ± 0.21	2.65 ± 0.22	2.69 ± 0.21	2.75 ± 0.24	2.72 ± 0.28	2.78 ± 0.18	2.70 ± 0.29	2.81 ± 0.26	2.60 ± 0.30
Placental weight (g)	428.54 ± 85.14	392.69 ± 54.67	469.51 ± 98.77	448.21 ± 92.22	471.41 ± 107.49	418.38 ± 63.32	419.08 ± 72.71	417.71 ± 83.58	420.67 ± 65.56

Data are presented as mean and standard deviation. Nw: maternal normal-weight; Ob: maternal obesity (BMI ≥ 30 kg/m^2^); Ob + DHA: obesity supplemented with DHA. ^a^*p* < 0.05 Nw vs. Ob, ^b^*p* < 0.05 Nw vs. Ob + DHA; Kruskal–Wallis test.

## Data Availability

The authors confirm that the data supporting the findings of this study are available within the article and its supplementary materials. Any additional information will be shared upon request to the corresponding authors.

## Additional Points

Our study is preliminary and presents some limitations. The small number of women in the group limited the statistical power of analyses. The low adherence to DHA supplementation probably reduced possible effects on receptor-associated molecular components. The inability to specifically target the circulating levels of IGF2, soluble receptor and inflammatory markers did not allow assessment of whether the receptor is cleaved. *Summary Sentence*. Placentas of male newborns delivered by women with pregestational obesity registered the increased levels of IGF2R which were normalized to the levels of healthy normal-weight women after DHA supplementation throughout the pregnancy.

## Supplementary Materials

Supplementary Materials Suppl Figure 1. Suppl Table 1.
