# Supplementary material for: DHA Supplementation during Pregnancy in Women with Obesity Normalizes IGF2R Levels in the Placenta of Male Newborns
Source: Int J Endocrinol. 2023 Jun 27;2023:1515033. doi: 10.1155/2023/1515033 (PMC10319466; doi:10.1155/2023/1515033)
Supplement: Supplementary Materials — Suppl Figure 1. Suppl Table 1. [file 1515033.f1.zip › Suppl Table 1 .pdf]

Suppl Table 1. Sequence of primers for Real Time PCR. **Ta**: Annealing temperature.

**Gen**	**Forward primer**	**Reverse primer**	**Ta (°C)**
ADAM17	TCCAGCAGCATTCGGTAAGA	TTTACCATCCACCACCACGA	56
IGF2	GTTGAGGAGTGCTGTTTC	AGGTGTCATATTGGAAGAAC	56
IGF2R	AGGTGAATAAGGAAGAAGAGAC	ACTGACTTGGTGGTAATATGG	56
PLAU	AGCAGAGACACTAACGACTTCAG	CTTACTCACACTTACACTCACAGC	56
β-*ACTIN*	AGAGCCTCGCCTTTGCCGATCC	GACGACGAGCGCGGCGATATC	62
GAPDH	TGTAGTTGAGGTCAATGAAGGG	ACATCGCTCAGACACCATG	60
YWHAZ	ACTTTTGGTACATTGTGGCTTCAA	CCGCCAGGACAAACCAGTAT	57
