# Supplementary figures and images for: DHA Supplementation during Pregnancy in Women with Obesity Normalizes IGF2R Levels in the Placenta of Male Newborns
Source: Int J Endocrinol. 2023 Jun 27;2023:1515033. doi: 10.1155/2023/1515033 (PMC10319466; doi:10.1155/2023/1515033)

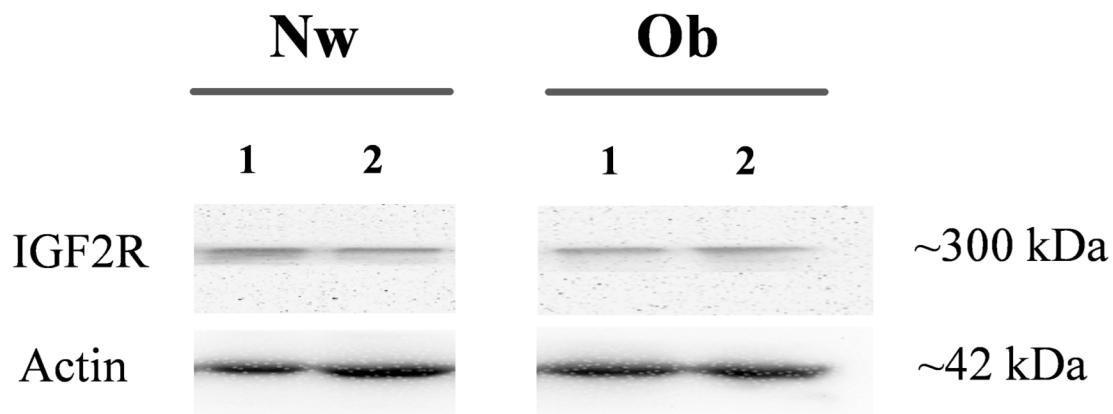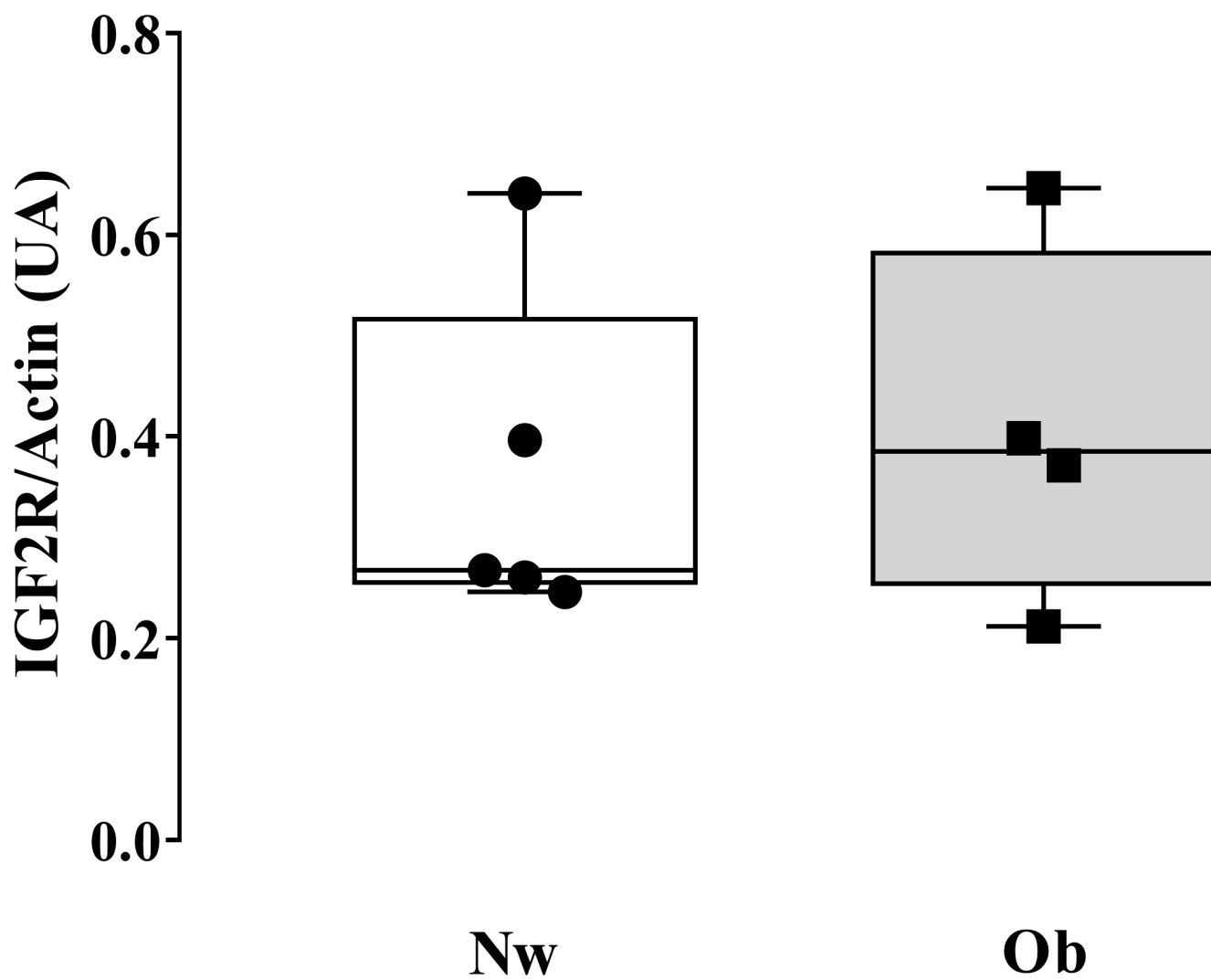

Supplement: Supplementary Materials — Suppl Figure 1. Suppl Table 1. [file 1515033.f1.zip › Fig Suppl 1.pdf]
